# Porcine Hepatic Response to Fumonisin B_1_ in a Short Exposure Period: Fatty Acid Profile and Clinical Investigations

**DOI:** 10.3390/toxins11110655

**Published:** 2019-11-10

**Authors:** Omeralfaroug Ali, Judit Szabó-Fodor, Hedvig Fébel, Miklós Mézes, Krisztián Balogh, Róbert Glávits, Melinda Kovács, Arianna Zantomasi, András Szabó

**Affiliations:** 1 Faculty of Agricultural and Environmental Sciences, Kaposvár University, 7400 Kaposvár, Hungary; kovacs.melinda@ke.hu (M.K.); szan1125@freemail.hu (A.S.); 2 “MTA-KE Mycotoxins in the Food Chain” Research Group, Hungarian Academy of Sciences, Kaposvár University, 7400 Kaposvár, Hungary; szabo.fodor.judit@gmail.com; 3 Research Institute for Animal Breeding, Nutrition and Meat Science, National Agricultural Research Center, 2053 Herceghalom, Hungary; febel.hedvig@atk.naik.hu; 4 Department of Nutrition, Faculty of Agricultural and Environmental Sciences, Szent István University, 2103 Gödöllő, Hungary; mezes.miklos@mkk.szie.hu (M.M.); balogh.krisztian@mkk.szie.hu (K.B.); 5 Autopsy Ltd., Telepes u. 42, 1147 Budapest, Hungary; glavits.robert.dr@gmail.com; 6 Department of Animal Science, University of Padova, Agripolis, Viale dell’Università 16, 35020 Legnaro, Padova, Italy; mania9123@gmail.com

**Keywords:** fumonisin B_1_, piglet, liver, lipids, blood serum, oxidation, clinical chemistry, histopathology, phospholipids

## Abstract

Scarce studies have investigated the impact of fumonisin B_1_ (FB_1_) on the hepatic tissue fatty acid (FA) profile, and no study is available on piglets. A 10-day in vivo experiment was performed on seven piglets/group: control and FB_1_-fed animals (diet was contaminated with fungal culture: 20 mg FB_1_/kg diet). Independent sample *t*-test was carried out at *p* < 0.05 as the significance level. Neither growth, nor feed efficiency, was affected. The hepatic phospholipid (PL) fatty acids (FAs) were more susceptible for FB_1_, while triglyceride (TG) was less responsive. The impact of FB_1_ on hepatic PL polyunsaturated fatty acids (PUFAs) was more pronounced than on saturated fatty acids. Among all PUFAs, predominant ones in response were docosapentaenoicacid (DPA) (↓), docosahexaenoic DHA (↓) and arachidonic acids (↑). This led to a higher omega-6:omega-3 ratio, whereas a similar finding was noted in TGs. Neither total saturation (SFA) nor total monousaturation (MUFA) were affected by the FB_1_ administration. The liver showed an increase in malondialdehyde, as well as antioxidant capacity (reduced glutathione and glutathione peroxidase). The plasma enzymatic assessment revealed an increase in alkaline phosphatase (ALP), while alanine transaminase (ALT), aspartate transaminase (AST), lactate dehydrogenase (LDH), and gamma-glutamyltransferase (GGT) were not influenced. The microscopic sections provided evidence of vacuolar degeneration of the hepatocytes’ cytoplasm, but it was not severe. Furthermore, the lung edema was developed, while the kidney was not affected. In conclusion, regarding FB_1_-mediated hepatotoxicity in piglets, the potential effect of slight hepatotoxicity did not compromise growth performance, at least at the dose and exposure period applied.

## 1. Introduction

Fumonisin B1 (FB_1_) can be regarded as one of the most important mycotoxins, due to its toxicity and carcinogenic mode of action [1,2]. FB_1_ can induce hepatotoxicity (proven in vitro and in vivo) and kidney cancer in mammals [3,4,5,6,7]. FB_1_ has a structure similar to the ceramide precursor sphinganine and therefore it inhibits the de novo ceramide synthesis (CerS); it catalyzes the acylation of sphinganine (Sa) and recycling of sphingosine (So) and interferes with the sphingolipid metabolism. This results in a higher concentration of intracellular Sa and other sphingoid bases in cells and tissues [8], which are proapoptotic, cytotoxic, and growth inhibitors [9]. The Sa:So ratio has been reported to increase in the plasma by FB_1_ exposure from a 3.7 mg FB_1_ + FB_2_/kg diet [10]. Also, it was found to increase from a 5 mg FB_1_/kg diet in several organs of the piglet, including the liver [11].

Pigs have been considered as the most relevant and sensitive animal model with a digestive system highly similar to the human [12,13]. Basically, the kidney and liver are very important organs in mycotoxicity experiments, since both are involved in FB_1_ (and metabolized forms) elimination and detoxification [14]. In swine, specifically, FB_1_ was found to exert development of pulmonary edema and hepatotoxicity [4], moreover, it has been suggested as the key player behind pulmonary fibrosis [15]. With regard to the liver, several studies reported the ability of FB_1_ in inducing histomorphological alterations with dose range of 3.7–190 mg/kg diet and exposure time range of 5–83 days, using different exposure methods: contaminated diet, orally gavaged, and intravenously [10,16,17,18,19,20,21]. Also, it induced clinical signs, such as respiratory distress [11]. On the other hand, assessment of the liver function relies on the serum biochemical levels, including the enzymatic matrix of alanine transaminase (ALT), aspartate transaminase (AST), alkaline phosphatase (ALP), lactate dehydrogenase (LDH), and gamma-glutamyltransferase (GGT). Moreover, sometimes the alteration in serum enzymatic matrix occurred coupled with higher total cholesterol level [18,22]. The negative effects of FB_1_ decrease after the regulation establishment, in which numerous regulations are available. Regulations are locally different, varying in the maximum recommended level of FB_1_ in the food/feed. The U.S. Food and Drug Administration (FDA) has published guidance level (20 mg/kg) as the maximum level for total fumonisins (FBs: FB_1_ + FB_2_ + FB_3_) in corn or its byproducts intended for swine nutrition [23]. In addition, the FDA states the contaminated corn should not exceed 50% of swine diet. Therefore, the total FBs in a complete diet should not exceed 10 mg/kg. On the other hand, in Europe, the European Commission (EC) has recommended maximally 5 mg FB_1_ + FB_2_ kg feed in all complementary and complete feedstuffs for swine [24]. 

The cellular fatty acid profile is acknowledged as a useful biomarker to monitor disease status. To date, it is well known that the disruption for the membrane lipid profile is FB_1_ toxicity mechanism, as numerous in vivo and in vitro; relevant literatures are available and well documented on rodents, such as those by Gelderblom et al. [25,26,27], Riedel et al. [5,6], Burger et al. [7,28], Szabó et al. [29,30,31], and to a lesser extent on rabbits [32,33]. However, in many cases, in vivo studies of FB_1_ altered the lipid metabolism in the rat which displayed different patterns than in in vitro studies. FB_1_ was found to interfere with the metabolism of sphingolipids and ceramides-subjective lipid regulatory enzymes and was found to induce lipid peroxidation [6,27]. Lipid peroxidation influences the lipid/FA composition of cellular membranes, as it highly depends on the degree of FA unsaturation in membranes. In addition, FB_1_ was suggested not only to modulate lipid profile integrity of the hepatocellular membranes by changing the FA composition and enzyme activities, but also through modifying its membrane microdomains [7]. Systematically, such changes in the intracellular and extracellular levels of lipid mediators alter the expression and activity of signaling and regulatory pathways that control physiological processes critical for cell growth, differentiation, and normal cell function [34]. In this regard, FB_1_ has been suggested to induce cancer development through modulating the membrane integrity and lipid raft of cells [5,7].

Despite a recent study illustrating that 12.2 mg FBs/kg diet developed hepatotoxicity in piglets after 28 days of exposure [10], it is interesting to study the sub-acute effect of FB_1_ on the liver. From this point of view, earlier reports which applied 20 mg FB_1_/kg diet [18,21] on piglets did not provide alteration in the membrane profile of the liver, in connection with its hepatotoxicity. Monitoring alterations in lipids may assist for better understanding of FB_1_ toxicity mechanism of action. The pathophysiology mechanism of FB_1_ in the piglets’ liver is not yet well understood. The pig was our animal model in this study, with a primary focus on the liver. Thus, this study aimed to investigate the effect of orally administrated FB_1_ on the FA profile of membrane phospholipids (PLs) and that of tissue triglycerides (TGs) from the hepatic tissue of weaned piglets exposed to a diet contaminated with fungal culture (20 mg FB_1_/kg diet) for 10 days. Furthermore, the study seeks to illustrate the hepatotoxicity status at the shorter exposure period through investigating the clinical chemistry and histomorphological changes.

## 2. Results

### 2.1. Body, Organ Weight, Feed Intake, and Its Utilization Efficiency

It is worth highlighting that during the study period no mortality case was found. Regarding the results in Table 1, for the liver weights no significant difference was detected among the study groups (control and FB_1_-fed group), even when expressed as relative weight of the total body weight. Similarly, the body weight, absolute weight of other organs (kidney, spleen, lung, and heart), and feeding parameters were not significantly affected by FB_1_.

### 2.2. Fatty Acid Profile of the Hepatic Phospholipids

When evaluating the hepatic PL FA (Table 2) composition, among all saturated FAs (SFAs), only lignoceric acid (C24:0) decreased to FB_1_ exposure (*p* < 0.01). Unsaturated FAs (UFAs) were more responsive, as compared to SFAs. Proportions of the C18:3n-6 (γ-linolenic acid, *p* < 0.05) and C20:4n-6 (arachidonic acid, *p* < 0.05) were significantly higher in FB_1_-fed piglets, while both of the C22:5n-3 (docosapentaenoic acid, DPA; *p* < 0.001) and C22:6n-3 (docosahexaenoic acid, DHA; *p* < 0.01) proportions were lower. Consequences of DPA and DHA proportional reduction are reductions in total polyunsaturation (PUFA; *p* < 0.05) and omega-3 FAs (*p* < 0.001). In contrast, significant increases were detected in total omega-6 FAs (*p* < 0.05) and n-6:n-3 ratio (*p* < 0.001). Total monounsaturation (MUFA) and total saturation (SFA) were not altered by FB_1_ feeding. From the calculated indices, unsaturation index (UI) and average fatty acyl chain length (ACL) decreased in the FB_1_-treated group (*p* < 0.05).

### 2.3. Fatty Acid Profile of the Triglycerides from Hepatic Tissue

In the hepatic TG FA profile (Table 2), C18:0 (stearic acid) proportion increased significantly (*p* < 0.05) in the FB_1_-fed group, as well as that in C20:4n-6 (arachidonic acid, AA; *p* < 0.01). The omega-6:omega-3 FA ratio increased significantly (*p* < 0.05) in piglets exposed to FB_1_. This increase was combined with a higher proportion of arachidonic acid, being the only responder FA (higher proportion) among all UFAs. No alterations were detected in the other calculated indices.

### 2.4. Lipid Peroxidation and Antioxidant Parameters

**Figure 1:**
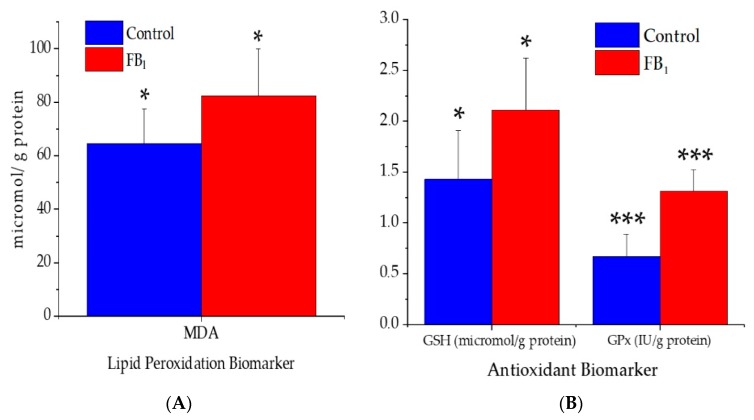
(**A**) Lipid peroxidation and (**B**) antioxidant biomarkers of the hepatic tissue of control and FB_1_-fed animals (data represent mean ± SD, *n* = 7 per group). * *p* < 0.05; *** *p* < 0.001; MDA, malondialdehyde; GSH, reduced glutathione; GPx, glutathione peroxidase.

Based on the results of the oxidative stress assessment parameters obtained from the liver, significant alterations were noticed (Figure 1). The tissue concentration of malondialdehyde (MDA) increased significantly (*p* < 0.05) due to FB_1_ administration. In a similar manner, the activity of glutathione peroxidase (GPx) increased (*p* < 0.001), as well as the level of the reduced glutathione (GSH, *p* < 0.05).

### 2.5. Serum Biochemical Parameters

**Figure 2:**
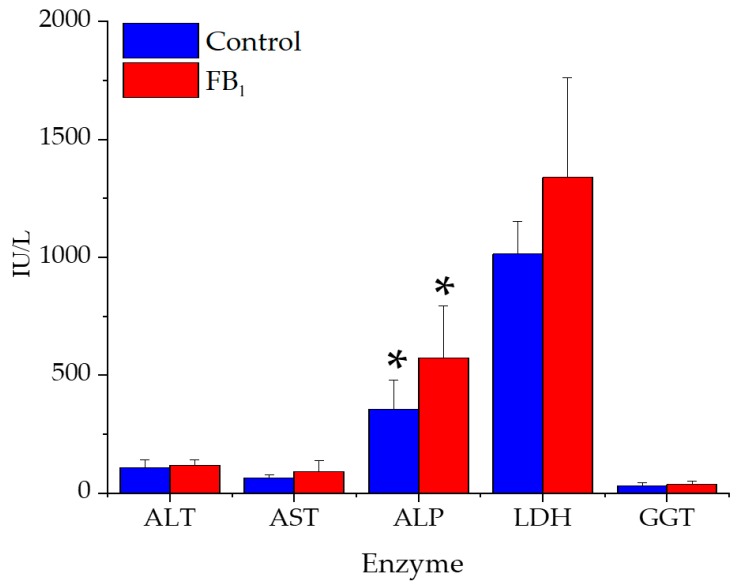
Alteration in serum enzymes for control and FB_1_-fed groups (20 mg FB_1_/kg feed) during the 10-day feeding period (data represent mean ± SD, *n* = 7 per group). * *p* < 0.05; ALT, alanine transaminase; AST, aspartate transaminase; ALP, alkaline phosphatase; LDH, lactate dehydrogenase; GGT, gamma-glutamyltransferase.

The blood serum biochemical parameters ALT, AST, ALP, LDH, and GGT can be seen in Figure 2. 

Only alkaline phosphatase (ALP) showed a significant activity increase after 10 days of toxin administration, while activity differences of the ALT, AST, LDH, and GGT in the blood serum were insignificant. Similarly, to the latter enzymes, concentrations of total protein, albumin, cholesterol, and bilirubin were not different (Table 3).

### 2.6. Histopathological Results

The histopathological assessment of the hepatic and lung tissues (see Table 4) has revealed changes in FB_1_-fed animals. In regard to the renal and spleen tissues, no lesion was found in any of the animals.

**Figure 3:**
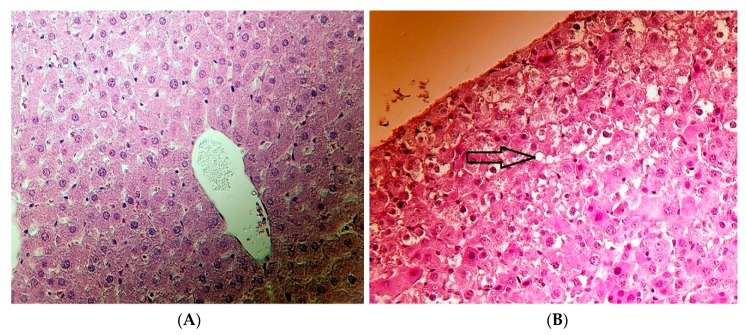
(**A**) Healthy (no lesion detected) liver of the control and (**B**) vacuolar degeneration of the hepatocytes (arrow) of weaned piglets after 20 mg FB_1_/kg diet exposure for 10 days (hematoxylin-eosin, 400×).

In some FB_1_-fed animals (5 piglets), slightly different degrees of vacuolar degeneration of the hepatocytes’ cytoplasm were observed (Figure 3). Those lesions were considered to be mild in severity (not substantially affecting the organ function) and were prone to recovery (healing).

In the lung, some animals fed FB_1_ have shown histopathological alterations. Most findings were mild edema in the lung interstitium (3 animals) and cavity of some alveolar groups (3 animals), which was associated with the effect of the FB_1_ toxin fed.

## 3. Discussion

The research area of FB_1_-induced modulation of tissue FA profile is receiving attention. Numerous studies reported the interfering ability of FB_1_ with FAs, PLs, and sphingolipids, but their exact role and extent is still unknown, especially in piglets. 

### 3.1. Growth, Feed Intake, and Organ Weights

**Figure 4:**
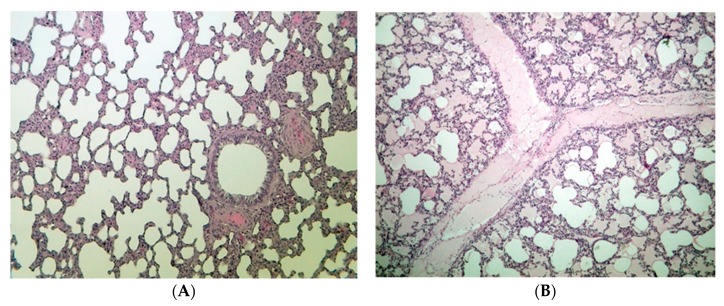
(**A**) Healthy (no lesion detected) lung of the control, and (**B**) alveolar and interstitial edema of the lung of weaned piglets after 20 mg FB_1_/kg diet exposure for 10 days (hematoxylin-eosin, 100×).

The administration of 20 mg FB_1_/kg diet for 10 days resulted in no mortality case. Mortality caused by FB_1_ is highly associated with high doses (FB_1_ >100 mg/kg diet) and/or a longer exposure period (>8 weeks and above) [35]. Furthermore, it associates with acute porcine pulmonary edema [36]. In addition, the growth, feed intake, and efficiency were not different between groups. The body weight highly relied on the feed intake, which was influenced by feed palatability. The authors suggest that the artificial FB_1_ contamination in our setting did not alter the feed palatability. Regarding the body weight gain and feed consumption, similar results to our findings were observed in weaned piglets fed 10, 20, and 40 mg/kg FB_1_ for four weeks [15]. Furthermore, 9 mg FB_1_/piglet/day for four weeks did not induce alteration in the production performance: growth, organ weights, and feed intake [10,37]. FB_1_ at 10–15 mg/kg diet is able to delay the piglet sexual maturity during longer exposure period at 24 weeks [38]. In animals, FBs (mostly FB_1_) typically damage the liver and kidneys (in a species-dependent manner), decrease body weight gain, and increase mortality rates [39]. In our setting, FB_1_ did not affect the kidney weight, which is interesting since its elimination happens via renal filtration [14], and partly through feces. Our results are in full agreement with the study of Souto et al. [37] and partially with results of Andretta et al. [40], in which FBs did not affect the weight of the kidney, spleen, and heart, but increased the relative weight of the liver and lung. The probable weight alteration of the liver was based on the hypothesis that FB_1_ provides slight hepatotoxicity in swine and rats [12,25]. The onset of hepatotoxicity was proven (mild and not severe), thereby no alteration was noticed in body weight, feed efficiency, and liver weight. With regards to the lung, FB_1_ has a rather strong effect on pig lung [4], thereby edema development has been hypothesized. A very slight pulmonary edema was proven in this study (Table 4, Figure 4), but since this has been reported earlier by Haschek et al. [4], we avoided discussing this in detail. However, our findings provide support to the statement of Haschek et al. [4] that lung and liver of swine are sensitive organs to FB_1_.

### 3.2. Fatty Acid Profile of the Hepatic Phospholipid FA Profile

A few in vivo and in vitro studies have investigated the effects of FB_1_ on lipid metabolism in hepatic tissue, mainly in rodents. A novelty of this study is adding value to the piglet-relevant literature available. Results have shown numerous alterations in the hepatic PL FA profile, although they were not intense/severe. Only a minor modification was detected in SFAs, where the lignoceric acid proportion decreased. Lignoceric acid is a member of the long chain SFA group; an important component of sphingomyelins (SPH). The biosynthesis of SPH is relying on ceramides production, a key intermediate of sphingolipid metabolism and major precursor of long chain FA and complex sphingolipids [41,42]. Therefore, such a proportional reduction of lignoceric acid in the PLs is indirectly referring to the inhibition in the production of ceramide and sphingomyelin, a characteristic of the FB_1_ mode of action.

FB_1_ seems to attack hepatic PLs more intensely, as compared to TGs (Table 2). Essential FAs are commonly esterified to the *sn*-1 position and occasionally to the *sn*-2 position of the PLs. Within omega-3 FAs, except for DPA and DHA (decreases), none of their ALA long chain derivatives were modified. DPA and DHA cumulative effects were more visible in the total n-3 FA proportion, decreasing significantly. In rats exposed to 50 mg/kg feed FB_1_ for five days, omega-3 FAs were found to decrease in the hepatic total PLs [28]. A similar reduction was reported by Szabó et al. [33] in the hepatic mitochondrial membrane of rabbits exposed to 10 mg/kg feed dose of FB_1_. Equal ALA proportion between groups is related to resemble feed consumption, since its only source is the diet. Accordingly, alteration in ALA derivatives (namely the DPA and DHA) must be with high probability a toxin effect. Moreover, reactive oxygen species attack FAs according to their degree of polyunsaturation [43], in which omega-3 FAs are more prone. 

The omega-6 FAs in the membrane have different roles from the omega-3 ones, although they may have common indications. C18:2n-6 (linoleic acid or LA, essential FA) equality among all groups in PLs and TGs may indirectly represent the identical diet uptake, similar to the ALA finding. FB_1_ exposure has increased the proportions of γ-linolenic and arachidonic acids (C20:4n-6). There are contradicting literatures [25,26] from the prolonged FB_1_ feeding studies on rats. Several studies reported the increase of arachidonic acid, such as Burger et al. [28], in rats treated for 21 days with 250 mg FB_1_/kg feed dosage. Authors supposed a shift towards prostanoid synthesis of the E2 series and added that alterations in the phosphatidyl-ethanolamine FA composition and arachidonic acid proportion in the plasma membrane could alter growth regulatory factors and cell receptors in lipid rafts. Furthermore, some adverse effects (i.e., cancer development, tumor angiogenesis, cell adhesion, and an increase in DNA synthesis) have been correlated with high proportions of arachidonic derived eicosanoids [44]. Therefore, the proportional increase of arachidonic acid in the piglet liver may be likewise a targeted accretion of the root fatty acid component for eicosanoid synthesis. 

The higher γ-linolenic and arachidonic acids’ proportions have thus increased the proportion of total omega-6 FAs, alongside with the increase of omega-6:omega-3 ratio. This ratio is a biological marker for disruption of enzymes that regulate lipid metabolism. In the study of Burger et al. [28] on rats, the omega-6:omega-3 ratio increased with toxin administration, which is similar to ours in piglets. Most probably, the lipid peroxidation process is the key player behind this result through oxidative stress propagation, an indirect toxin effect. For this reason, our study revealed that the porcine liver is a sensitive organ to FB_1_, and its toxicity can be linked with its membrane profile.

Regarding FA indices, a reduction was noted for the UI as a result of depletion in total PUFA proportions of FB_1_-fed piglets. As a consequence, ACL decreased, highly influenced by the reduction of DPA and DHA. The reduction in PUFA and UI may refer to a more rigid cell membrane, as a protective way against FB_1_, likely by manipulating membrane receptors and enzymes activities that are involved in the biosynthesis of proteins, lipids, and sterols. However, the MUFA level was also not responsive for the treatment applied. These insignificant results were unexpected, since relevant studies illustrated the increase in total saturation and monounsaturation is a way to increase the membrane rigidity, a resistance mechanism against oxidative stress [6].

### 3.3. Fatty Acid Profile of the Hepatic Triglycerides

Only a few modifications were observed in the hepatic TGs. Compositional changes of TGs are seldom reported and are generally referring to energy metabolism. TG stores have fewer other functions than energy supply and are thus mostly reflecting the need for specific FAs at an extra-hepatic site. Once we only registered minor changes, our TG dataset may be handled as secondary data. Similar to PL results, the omega-6:omega-3 ratio increased, as well as that in arachidonic acid. Despite TGs major role to provide energy, their compositional modifications were linked to alterations in the physical properties of cellular membranes [45]. The main reason behind this is that they are incorporated into the lipid bilayer and assist the maintenance of cell membranes.

### 3.4. Lipid Peroxidation and Antioxidant Parameters

Oxidative stress is a condition produced by free radical accumulation that is not entirely eliminated by antioxidants. Studies demonstrate the ability of FB_1_ in the oxidation stress induction via generation of reactive oxygen species (ROS). ROS over-production by mitochondrial indicates damage on its membrane, associated with the transient activation of cytosolic phospholipases A2 (cPLA_2_) [46]. Activity of cPLA2 is influenced by SPH concentration [47], in which FB_1_ mechanism of action involves SPH disruption. Within a specific time-frame, ROS attack within the cell will contribute to its depletion and deterioration of its biomolecules and favor cell death conditions through stimulating certain stress-sensitive signaling pathways (e.g., nuclear factor κB, p38MAPK, and c-Jun N-terminal kinase) [48,49]. Lipid peroxidation (lipid attacked by ROS) is a consequence of FB_1_ toxicity mode of action, whereas MDA level is acknowledged as a reliable biomarker for FA peroxidation and cell membrane damage [50]. MDA is cytotoxic and results from the terminal phase of FA peroxidation, majority three double bond containing FAs and with a considerable amount less than three double bond FAs [51,52]. In this study MDA level was significantly modified, indicating that the liver was undergoing oxidative stress. Consequently, alterations in its membrane lipid profile are possibly linked with oxidative stress. 

When the MDA level was increasing, the free radical scavenger (GSH) and activities of GPx enzymes were stimulated as well. The increase of GSH and GPx in the hepatic tissue were unfamiliar since they were decreasing in rabbits, rats, and pigs exposed to FB_1_. In the study of Szabó et al. [33] on weaned rabbits exposed for four weeks to 10 mg FB_1_/kg dietary, reduction in the GSH and GPx of the plasma was present. Similar reductions in the GSH level were reported in the rat hepatic tissue [29], blood, and hemolyzed red blood cells (RBCs) of piglets [14]. In our study, no reduction was observed in levels of GSH and GPx, we suggest that oxidative stress and its derived byproduct production were less pronounced to compromise (decrease) compounds involved in the antioxidant defense mechanism. 

The steady state level of cellular GSH builds on the equilibrium between production and consumption, extrusion and reduction in the cell, and oxidation and bond forming [53]. It is well known that GSH biological roles are not exclusive on the antioxidant defense mechanism. However, the GSH reinforced in hepatic tissue was achieved by increasing the activity of GPx enzymes, and thus eliminating the mitochondrial free radicals and byproducts of lipid peroxidation.

### 3.5. Serum Clinical Chemistry

In mammals, albumin represents most protein of the blood plasma [54], almost 60% of the total. Albumin levels were not different between groups. This refers to the non-compromised hepatic protein synthesis. It means no alteration at the glomerular permeability as well. This probably means the kidney was functioning well without nephrotoxicity. However, it is interesting to note that the concentration of albumin was in correlation with histopathological assessment, although its concentration was not statistically different between groups. Probably, the duration of exposure was the key player behind such a finding, since the mild hepatotoxicity was confirmed. 

FB_1_ administration has not altered the serum lipid total cholesterol concentration. This is unfamiliar with what was observed in piglets gavaged 1.5 mg FB_1_/kg BW (equal to 25–30 mg FB_1_/kg diet) for nine days [22], and also in piglets fed 12.2 mg FBs for 28 days [10]. In addition, this was observed in rats [55], due to the negative regulation of liver X receptors, the nuclear receptor family regulates the expression of genes involved in cholesterol and lipid homeostasis [56]. The reason behind this insignificant result in our study might be that the response is species-specific, thereby piglets had a different reaction than rats. In addition, the consumed FB_1_ (93 mg/piglet) during the exposure period was lesser than that exposed in the study of Loiseau et al. [22]. Furthermore, Loiseau et al. [22] used oral gavage of a single FB_1_ dose on a daily basis, and total daily dose was received at a specified moment. This is not similar to the case when the animal is exposed to FB_1_ through diet, as animals receive a similar dose amount at a longer period, depending on the feeding system. Moreover, the longer exposure period (almost 300% as compared to ours) in a study of Terciolo et al. [10] was able to alter the serum cholesterol, which was not similar to our findings due to the shorter exposure period. 

Hepatotoxicity is generally characterized by alterations in organ weight and serum enzyme activities [22]. The serum bilirubin was not responsive for FB_1_, referring to normal physio-activities of the liver and pancreas. However, further investigations are needed to confirm this hypothesis. The ALT, AST, GGT, and LDH were not statistically altered by toxin exposure. In a study on piglets [21] at the same FB_1_ level, but longer exposure period (two weeks), neither ALT, nor AST differed from the control. In pigs, ALP is a strongly responsive enzyme to FB_1_ toxicity [57]. Such an increase was toxin-impact, as this has been proven in pigs [16,18,19,58], and male and female Sprague Dawley rats [59]. Once measured enzymes were not responsive for the treatment, except ALP, it is a likely indicator for the commencement hepatotoxicity phase and/or generalized bone dysfunction. 

When rats were exposed to 90 mg FB_1_/kg body weight for 21 days, hepatotoxicity was found to be sufficient to trigger the mineral balance leading into alterations in bone metabolism and its mechanical endurance, although bone mass was not affected [60]. The role of ALP in bone development has been well acknowledged [61], therefore, we suggest that alteration in ALP activity, as induced by FB_1_ has a role in the impairment of mineral homeostasis. However, authors assumed that the dysfunction in mineral metabolism was absent (serum ions were unchanged, data not shown), under the present slight/initial hepatotoxicity. Accordingly, possible mild hepatotoxicity action was the key factor behind ALP induction and no other enzymes. Another possible scenario is FB_1_ has altered the intestinal structure [62] of the enterocytes (not tested here). 

### 3.6. Histopathological Investigation

Hepatotoxicity induced by FB_1_ is well documented in the relevant literature, mostly tested in rats while scarcely on swine, horses, and rabbits. From the literature in swine, FB_1_ induces pulmonary edema and provides slight hepatotoxicity [4,10,12]. Pigs are highly susceptible to FB_1_-induced hepatotoxicity, regardless of the administration method, orally or intravenously [16]. In the present investigation, the most striking modifications seen in the liver of piglets exposed to 20 mg FB_1_/kg diet are the vacuolar degeneration of hepatocyte cytoplasm. This finding was markedly categorized as a mild to moderate effect, in agreement with the findings of Dilkin et al. [20] and Kovács et al. [21]. The vacuolar degeneration of hepatic cell cytoplasm is highly associated with disturbance of cellular water or lipid metabolism, indicative of an exertion of the cell’s metabolic and/or detoxification activity. However, no other change was detected in the liver, which is not similar to earlier reports [16,18]. Such variance might be mainly attributed to the relatively low dose applied in our study. 

Cholestasis is a condition involving interruption of the bile production and/or secretion [63], whereas it is associated with vacuolar degeneration. Analyzing Table 3, the unchanged serum bilirubin level is indirectly indicating the absence of hepatocellular-cholestasis, and therefore our microscopic findings do not refer to cholestasis. It has been reported that FB_1_ can modify protein biosynthesis [64], which may imply degenerative alterations in the tissue. This finding was not observed here, since albumin concentrations were equal among the experimental groups, meaning no protein synthesis fallback. We suggest that oxidative stress modulation in lipid metabolism was the indirect player/factor behind FB_1_ which induced start-up hepatotoxicity and developed vacuolar degeneration, a result of stress and not inflammation since the liver weight was unchanged, although it is not a precise biochemical indicator. In this regard the Sa:So biomarker (not tested) may assist in clarifying the histopathological lesion observed, and also other measured parameters. In summary, the histopathological assessment indicated mild status, which in general provides evidence for the onset necrotic process, in which it is reversible (healing).

Interestingly, histopathological assessment of the renal tissue has revealed no intergroup difference, referring to absent nephrotoxicity. Such a result means the renal tissue is less sensitive to FB_1_ than the liver in piglets. This result is not consistent with the recent published data, even at a lower FB_1_ level 12 mg FBs/kg diet [10]. Most probably the longer exposure period (as compared to ours) played an important role in the development of kidney lesions. A similar finding to our result was reported by Gumprecht et al. [20], when 20 mg FB_1_/kg diet for four days did not develop a microscopic lesion in the kidney, only in the lung and liver. This is rather different from the sensitivity of rats, where the kidney is the most relevant organ for FB_1_ toxicity [30,65].

## 4. Conclusions

From the study point of view, the orally administrated 20 mg FB_1_/kg diet induced the commencement of hepatotoxicity in piglets. Therefore, this study suggests that the applied dose (20 mg FB_1_/kg diet) is not safe for weaned piglets two months age and with a body weight below 16 kg, even under a short exposure period (10 days), although the production performance was not compromised. This study illustrates the sub-acute negative effects of FB_1_ in a shorter period on the liver, as compared to earlier reports. Alterations of membrane lipid profile could be due to the destruction of UFAs and/or disturbance of FA desaturase enzymes. In addition, alterations of PLs can be due to the destruction of the PL domain and/or disruption of CerS. Apparently, further investigation on Sa:So ratio would be important for finer clarification, since they are efficient biomarkers for assessing CerS disruption and even toxicity status of the liver, induced by FB_1_. 

In general, our results may facilitate to better perceive the modulation in lipid sites associated with cellular damage, induced by FB_1_. However, this is the first in vivo study reporting the lipid profile alterations as a result of FB_1_ impact on the hepatic tissue of weaned piglets. The study has handled total PL and TG, whereas investigating PL subclasses is more worthy. Furthermore, a clear visualization requires a bigger population size (i.e., more piglets). Therefore, further investigations are necessary to determine FA involvement in hepatocellular damage of pigs, which can be performed by handling the different phospholipid subclasses, applying multiple doses, and exposure periods.

## 5. Material and Methods

### 5.1. Ethical Allowance

The experiment was carried out according to the Hungarian Animal Protection Act, in compliance with the relevant EU rules. The experimental protocol has been authorized by the Food Chain Safety and Animal Health Directorate of the Somogy County Agricultural Office, under permission number XV-I-31/1509-5/2012 (approved on 27 November 2012).

### 5.2. Experimental Design and Nutrition

Fourteen weaned barrows of the same genotype (Landrace X Yorkshire), weighing 13–14 kg (50 days of age) were used in the experiment. The piglets were weighed and then divided into two groups: an experimental group (FB_1_-fed) and a control (*n* = 7/group). Animals were kept individually during the trial. Feed was given twice a day, in two equal portions, and the amount of feed not consumed by the animals was measured back; drinking water was available ad libitum via automatic drinkers.

Animals were kept for 7 days as an adaptation period, while the duration of the feeding trial was 10 days. Experimental animals were fed a basic ration of a nutrient composition corresponding to their age, containing feed of identical proximate component (Table 5). After this period, a *Fusarium verticillioides* fungal culture (strain MRC 826, for production details see: [66]) was added to the diet and homogenized. This contaminated diet was fed to the FB_1_-fed group, so as to provide a daily FB_1_ intake of approximately 10 mg FB_1_/animal/day (equivalent to 0.5 kg feed consumption/animal/day). 

The fungal culture typically contained 3.4 mg FB_1_/g, and small quantities of less toxic compounds FB_2_ and FB_3_, 0.6 and 1 mg/g, respectively. The mycotoxin concentration of the control and the experimental feed was determined with LC-MS (Shimadzu, Kyoto, Japan) with 3 µg/kg limit of detection (LOD) for FB_1_. The diet fed to the control group did not contain detectable amounts of FB_1_ (below the LOD), while deoxinivalenol, zearalenone, and T-2 toxin were well-controlled and absence was confirmed. 

Animals were kept with only drinking water available (without feed) 12 h before the scarification. At the end of the trial, the piglets were euthanized and exsanguinated after sedation (euthanyl-pentobarbital sodium, 240 mg/mL) and liver and blood were sampled for analysis.

### 5.3. Lipid Analysis of the Hepatic Tissue

The liver sample (after storage at −20 °C) and the feed were homogenized (IKA T25 Digital Ultra Turrax, Staufen, Germany) in 20-fold volume of chloroform-methanol (2:1, *v*:*v*) and total lipid content was extracted according to Folch et al. [67]. Solvents were ultrapure-grade (Merck Sigma-Aldrich, Schnelldorf, Germany) and 0.01 % *w*:*v* butylated hydroxytoluene was added to prevent fatty acid oxidation.

For the separation of lipid fractions (TG and PL), extracted total lipids were transferred to glass chromatographic columns, containing 300 mg silica gel (230–400 mesh) for 10 mg of total lipids [68]. Neutral lipids were eluted with 10 mL chloroform for the above fat amount, then 15 mL acetone: methanol (9:1, *v*/*v*) was added, while 10 mL pure methanol eluted the total phospholipids. This latter fraction was evaporated under a nitrogen stream and was trans-methylated with a base-catalyzed NaOCH_3_ method [69].

Fatty acid methyl esters were extracted into 300 μL ultrapure n-hexane for gas chromatography, which was performed on a GCMS-QP2010 Plus equipment (AOC 20i automatic injector), equipped with a Phenomenex Zebron ZB-WAX Capillary GC column (30 m × 0.25 mm ID, 0.25 micrometer film, Phenomenex Inc., Torrance, CA, USA). Characteristic operating conditions were as follows: injector temperature: 270 °C; helium flow: 28 cm/sec. The oven temperature was graded from 80 to 205 °C: 2.5 °C/min, 5 min at 205 °C and from 205 to 250 °C: 10 °C/min, 5 min at 210 °C. The makeup gas was nitrogen. To identify the individual FAs, an authentic external FA standard (37 Component FAME Mix, Merck Sigma-Aldrich, Cat. No.: CRM47885) was used. Fatty acid results were expressed as weight % of total fatty acid methyl esters.

Unsaturation index was defined as the number of double bonds in 100 fatty acyl chains. From the FA results, UI was calculated as: UI = ((1 × Σ monoenoic FA) + (2 × Σ dienoic FA) + (3 × Σ trienoic FA) + (4 × Σ tetraenoic FA) + (5 × Σ pentaenoic FA) + (6 × Σ hexaenoic FA)) [70]. The average fatty acyl chain length was calculated from the multiplication of the chain length values and the respective proportion of each FA.

### 5.4. Determination of Lipid Peroxidation and Antioxidant Capacity

Samples of hepatic tissue were stored at −82 °C before analysis. Lipid peroxidation was assessed with the determination of MDA levels with 2-thiobarbituric acid method [71] in the 10-fold volume of tissue homogenate in physiological saline. The amount of GSH and GPx activity was measured in the 10,000 g supernatant fraction of tissue homogenate. The quantification of the GSH was measured as non-protein thiols by Ellmann’s reagent [72], while the activity of GPx was according to Lawrence and Burk [73]. GSH concentration and GPx activity were calculated to protein content of the 10,000 g supernatant which was measured by the Folin-phenol reagent [74]. In all instances the color was measure with UV–Vis spectrophotometry in 10 mm pathway optical glass cuvettes.

### 5.5. Serum Clinical Chemistry Analysis

The different clinical parameters of serum-total protein, albumin, creatinine, glucose, urea, and the total cholesterol concentrations and the activity of aspartate aminotransferase (AST) and alanine aminotransferase (ALT) were determined in a veterinary laboratory (Vet-Med Laboratory, Budapest, Hungary), using Roche Hitachi 917 Chemistry Analyzer (Hitachi, Tokyo, Japan) with commercial diagnostic kits (Diagnosticum LTD., Budapest, Hungary).

### 5.6. Histopathological Analysis

Immediately after piglets were sacrificed, the liver and lung samples were collected and stored in 10% neutrally buffered formalin and embedded in paraffin for the histopathologic investigation, under light microscope. Regarding the microscope analysis, the microtome slides of 5 micron (µ) were prepared and stained with hematoxylin-eosin.

The main pathological alterations were described and scored, according to their extent and severity as follows: (-) = no alteration; 1 = slight/small scale/few; 2 = medium degree/medium scale/medium number.

The histopathological analysis was carried out according to the Act #2011 (03.30) of the Hungarian Ministry of Agriculture and Rural Development and was in accordance with the ethical guidelines of the Organization for Economic Cooperation and Development (OECD) Good Laboratory Practice for Chemicals (1997).

### 5.7. Statistical Evaluation

All data were tested for normality (Shapiro–Wilk test); after this, control and FB_1_-fed groups’ means were compared with an independent sample *t*-test, using IBM SPSS for Windows version 20 (2009). However, for group differences, the calculating probability (*p*-value < 0.05) used as the significance level.

## Figures and Tables

**Table 1:** The somatic and feeding parameters of the control and fumonisin B_1_ (FB_1_)-fed piglets.

Somatic Traits	Control			FB_1_		
	Mean	±	SD	Mean	±	SD
BW initial (kg)	13.3	±	1.90	13.1	±	1.60
BW final (kg)	15.9	±	2.40	15.8	±	1.80
DBWG (g)	266	±	66.3	269	±	33.3
FC (kg/10 days)	4458	±	1022	4650	±	443
FCR (g diet/g BWG)	1.69	±	0.13	1.74	±	0.20
Liver (g)	359	±	78.9	358	±	56.2
Kidney (g)	85.2	±	9.99	81.3	±	11.4
Spleen (g)	38.3	±	4.29	36.1	±	5.16
Lung (g)	198	±	34.4	189	±	37.7
Heart (g)	99.2	±	14.9	106	±	12.5

BW, body weight; DBWG, daily body weight gain; FC, feed consumption; FCR, feed conversion ratio.

**Table 2:** Fatty acid profiles of the total phospholipid (PL) and triglycerides (TGs) from the hepatic tissue for control and FB_1_-fed piglets.

Fatty Acids	Hepatic Total PL (%)							Hepatic Total TG (%)						
	Control			FB_1_				Control			FB_1_			
	Mean	±	SD	Mean	±	SD	Sig.	Mean	±	SD	Mean	±	SD	Sig.
C12:0	-	±	-	-	±	-	-	0.04	±	0.01	0.03	±	0.01	NS
C14:0	0.13	±	0.03	0.16	±	0.03	NS	0.35	±	0.14	0.26	±	0.09	NS
C15:0	0.13	±	0.07	0.17	±	0.11	NS	0.10	±	0.04	0.12	±	0.06	NS
C16:0	15.5	±	0.60	15.2	±	0.95	NS	12.3	±	2.25	11.1	±	0.70	NS
C16:1n-7	0.30	±	0.07	0.38	±	0.16	NS	0.56	±	0.30	0.38	±	0.14	NS
C17:0	0.72	±	0.23	1.09	±	0.65	NS	0.63	±	0.22	0.88	±	0.61	NS
C18:0	29.0	±	0.93	29.6	±	1.62	NS	26.6	±	1.88	28.6	±	1.11	*
C18:1n-9c	5.75	±	0.54	6.48	±	1.17	NS	8.17	±	2.83	6.62	±	0.92	NS
C18:2n-6c	20.6	±	1.04	20.7	±	0.75	NS	18.4	±	1.77	18.0	±	0.74	NS
C18:3n-6	0.14	±	0.03	0.18	±	0.05	NS	0.14	±	0.08	0.19	±	0.05	NS
C18:3n-3	0.21	±	0.05	0.17	±	0.03	NS	0.56	±	0.28	0.40	±	0.13	NS
C20:0	0.07	±	0.01	0.08	±	0.02	NS	0.13	±	0.06	0.11	±	0.01	NS
C20:1n-9	0.12	±	0.02	0.12	±	0.02	NS	0.15	±	0.11	0.20	±	0.08	NS
C20:2n-7	0.66	±	0.08	0.63	±	0.06	NS	0.70	±	0.10	0.64	±	0.05	NS
C20:3n-6	1.34	±	0.35	1.29	±	0.25	NS	1.29	±	0.38	1.33	±	0.25	NS
C20:4n-6	12.1	±	1.36	13.4	±	0.41	*	15.5	±	1.66	17.9	±	1.12	**
C20:3n-3	0.14	±	0.04	0.12	±	0.02	NS	0.21	±	0.06	0.20	±	0.04	NS
C20:5n-3	1.35	±	0.55	1.37	±	0.32	NS	1.44	±	0.57	1.54	±	0.26	NS
C22:0	0.03	±	0.00	0.04	±	0.01	NS	0.10	±	0.09	0.05	±	0.02	NS
C22:5n-3	3.04	±	0.14	2.38	±	0.29	***	3.13	±	0.34	2.89	±	0.25	NS
C22:6n-3	8.34	±	0.79	6.28	±	1.11	*	9.03	±	1.41	8.14	±	0.82	NS
C24:0	0.34	±	0.03	0.26	±	0.04	**	0.38	±	0.07	0.33	±	0.04	NS
SFA	46.0	±	0.75	46.6	±	0.77	NS	40.7	±	2.73	41.6	±	0.99	NS
UFA	54.0	±	0.75	53.4	±	0.77	NS	59.3	±	2.73	58.4	±	0.99	NS
MUFA	6.17	±	0.59	6.89	±	1.33	NS	8.88	±	3.21	7.19	±	1.10	NS
PUFA	47.8	±	1.07	46.5	±	1.39	*	50.4	±	4.92	51.2	±	1.32	NS
Omega-6	34.1	±	0.81	35.5	±	0.53	*	35.4	±	3.03	37.4	±	0.85	NS
Omega-3	13.0	±	0.91	10.3	±	1.35	***	14.4	±	2.09	13.2	±	1.05	NS
n-6:n-3	2.62	±	0.21	3.49	±	0.44	***	2.49	±	0.23	2.86	±	0.26	*
Odd Chain	0.85	±	0.29	1.26	±	0.76	NS	0.73	±	0.26	1.00	±	0.66	NS
UI	174.3	±	5.43	164.8	±	6.50	*	192.8	±	16.4	193.3	±	5.36	NS
ACL	18.5	±	0.05	18.4	±	0.08	*	18.6	±	0.15	18.6	±	0.06	NS

* *p* < 0.05; ** *p* < 0.01; *** *p* < 0.001; NS, not significant, *p* > 0.05; SFA, saturated fatty acids; UFA, unsaturated fatty acids; MUFA, monounsaturated fatty acids; PUFA, polyunsaturated fatty acids; n-6:n-3, omega-6:omega-3; UI, unsaturation index; ACL, average chain length.

**Table 3:** The liver associated serum biochemical parameters for control and FB_1_-fed piglets (20 mg FB_1_/kg feed).

Parameter	Control			FB_1_		
	Mean	±	SD	Mean	±	SD
Total protein (g/L)	55.4	±	3.64	52.6	±	3.41
Albumin (g/L)	34.0	±	1.82	32.7	±	3.15
Total cholesterol (mmol/L)	1.98	±	0.21	2.10	±	0.18
Total bilirubin (μmol/L)	2.44	±	1.50	1.10	±	0.83

**Table 4:** The histopathological alteration in the hepatic and lung tissues for control and FB_1_-fed animals individually (20 mg FB_1_/kg feed) after a 10-day feeding period.

Organ	Parameters	Control							FB_1_						
		Animal Number													
		1	2	3	4	5	6	7	1	2	3	4	5	6	7
Liver	Vacuolar degeneration	-	-	-	-	-	-	-	1	1	-	1	1	2	-
Lung	Alveolar edema	-	-	-	-	-	-	-	2	-	1	-	1	-	-
	Interstitial edema	-	-	-	-	-	-	-	-	-	-	1	1	-	-

(-) = no alteration; 1 = slight/small scale/few; 2 = medium degree/medium scale/medium number.

**Table 5:** The proximate and fatty acide (FA) composition of the basal feed.

Component	Diet
DM (%)	90.8
Metabolizable energy (MJ/kg)	14.8
Digestible energy (MJ/kg)	14.2
Crude protein (% of DM)	19.7
Ether extract (% of DM)	5.8
Crude fiber (% of DM)	3.2
Ash (% of DM)	5.1
FA weight % of total FA methyl esters	
C10:0	0.02
C12:0	0.03
C14:0	0.4
C15:0	0.05
C16:0	15.2
C16:1n-7	0.44
C17:0	0.15
C17:1n-7	0.07
C18:0	4.85
C18:1n-9	26.7
C18:1n11t	0.09
C18:2n-6c	49
C18:3n-3	0.23
C20:0	0.36
C20:1n-9	2.13
C20:2 n-6	0.1
C22:0	0.11

DM, dry matter; FA, fatty acid.
